# Thrombocytopenia and Bleeding in Chronic Kidney Disease: A Case of Acquired Von Willebrand Syndrome

**DOI:** 10.7759/cureus.65834

**Published:** 2024-07-31

**Authors:** Amin A Alamin

**Affiliations:** 1 Pathology, College of Medicine, Taif University, Taif, SAU

**Keywords:** von willebrand factor concentrates, desmopressin, bleeding, thrombocytopenia, chronic kidney disease, acquired von willebrand syndrome

## Abstract

Acquired von Willebrand disease is a rare condition with laboratory findings similar to the inherited type, which can be autosomal dominant or recessive. This case describes a rather rare clinical situation of a 65-year-old man with stage 4 chronic kidney disease who also had acquired von Willebrand syndrome (AvWS) with thrombocytopenia and bleeding.

The patient had a complaint of easy fatigability, easy bruising, and prolonged bleeding from small cuts. The patient’s initial laboratory workup included thrombocytopenia, which on further evaluation established the diagnosis of AvWS due to chronic kidney disease. More specific examination revealed reduced activity of the von Willebrand factor. The patient was managed with desmopressin and von Willebrand factor concentrates and there was a transient rise in platelet count and relief of symptoms of bleeding. This case underlines the importance of AvWS in any differential diagnosis of thrombocytopenia in patients with chronic kidney disease. This report aims to provide recommendations for early identification and management of AvWS to improve the outcome.

## Introduction

Acquired von Willebrand syndrome (AvWS) is a bleeding disorder that is caused by a deficiency in the von Willebrand factor (vWF), which plays a key role in the coagulation cascade [1]. vWF also plays a role in the transport of factor VIII in the circulation and prevents its degradation at an early stage. This syndrome is a group of diseases with diverse causes, which include lymphoproliferative diseases, autoimmune diseases, cardiovascular diseases, and hypothyroidism [2].

Although recent studies identified the relationship between AvWS and chronic kidney disease (CKD), the relationship is still not clear. [3]. As to why AvWS develops in patients with CKD, several hypotheses have been proposed; one of which is that CKD patients have elevated levels of both total and active vWF. Despite these elevated levels, the function of vWF can be compromised. Uremia can alter the structure and function of vWF, reducing its effectiveness in promoting platelet adhesion and aggregation. Consequently, these qualitative changes in vWF can lead to complex hemostatic abnormalities, such as impaired platelet adhesion and aggregation, increased bleeding tendency, heightened thrombotic risk, impaired fibrinolysis, and endothelial dysfunction [4], lower vWF synthesis because of endothelial dysfunction - a frequent complication of CKD that affects the ability of endothelial cells to synthesize vWF [5], and impaired vWF function due to uremia, a state characterized by accumulation of waste products in the blood that alters platelet function and affects the binding of vWF to platelets and hence the function of vWF [6].

The symptoms of AvWS are very nonspecific and can range from minor skin and mucosal bleeding to major bleeding episodes [7]. The laboratory workup usually shows reduced vWF activity and antigen levels, prolonged bleeding time, and thrombocytopenia of variable severity, which is similar to inherited von Willebrand disease [7]. It has been stated that the diagnosis of AvWS can be difficult due to the fact that symptoms and laboratory findings are similar to other coagulation disorders [8]. First, to make a diagnosis and eliminate other causes of bleeding, it is necessary to perform a detailed history, physical examination, and a series of diagnostic tests.

The issue of AvWS in patients with CKD can best be tackled through a combination of measures. Desmopressin (DDAVP) is a synthetic form of vasopressin and is commonly used to manage patients with AvWS at the initial stage. DDAVP causes the release of vWF from endothelial cells and the vWF level increases briefly and the hemostasis is enhanced [9]. Nevertheless, DDAVP efficacy in CKD patients may be reduced because of the reduced renal elimination and possible drug interactions [10].

## Case presentation

A 65-year-old man with stable CKD due to chronic use of nonsteroidal anti-inflammatory drugs presented to the outpatient hematology clinic with a two-month history of easy fatigability, easy bruising, and prolonged blood clotting from minor injuries. The patient had no history of hematological disorders, medication changes, or recent trauma.

Physical examination was remarkable for multiple ecchymoses on both upper and lower extremities, ranging from 2 to 5 cm. Abdominal and neurological exams were unremarkable, with no signs of chronic liver disease.

Preliminary investigations revealed thrombocytopenia, mild anemia, a normal white blood cell count, and a normal coagulation profile (Table 1).

**Table 1:** Laboratory parameters of the patient.

Parameter	Value	Reference range
Platelet count	60 x 10^9/L	150-450 x 10^9/L
Hemoglobin	13.5 g/dL	Male: 13.8-17.2 g/dL
White blood cells (WBC)	6.5 x 10^9/L	4.0-11.0 x 10^9/L
Estimated glomerular filtration rate (eGFR)	35 mL/min/1.73 m^2	>60 mL/min/1.73 m^2
Prothrombin time (PT)	12.5 seconds	11-13.5 seconds
Activated partial thromboplastin time (aPTT)	30 seconds	25-35 seconds
Von Willebrand factor antigen (VWF)	0.35 UI/mL	0.50-1.50 UI/mL
Von Willebrand factor activity	30%	50%-150%
Factor VIII activity	90%	50%-150%
Platelet function	85%	80%-120%

Further examinations revealed low vWF antigen levels and reduced vWF activity. Normal factor VIII activity and platelet function tests supported a diagnosis of isolated AvWS secondary to CKD. The diagnosis was confirmed by reduced vWF activity and typical CKD-related laboratory findings.

The patient received a single dose of intravenous desmopressin (DDAVP) at 3 mcg/kg over 30 minutes, resulting in an increase in platelet count to 80 x 10^9/L within 24 hours and cessation of bleeding. However, platelet counts returned to baseline within a few days, indicating a short-lived response to DDAVP. The patient was kept under observation for bleeding, and plans were made for potential repeat DDAVP administration or alternative medications if needed.

## Discussion

AvWS differs from inherited vWF disorders and other bleeding disorders due to its distinct etiology and clinical presentation. AvWS arises from underlying conditions like CKD, malignancies, or autoimmune diseases, which affect vWF function and levels. It is characterized by a disproportionate decrease in vWF activity relative to antigen levels and abnormal multimer patterns, reflecting the impact of these underlying conditions [11]. In contrast, inherited vWF disorders are congenital and categorized into types based on the nature of vWF deficiency or dysfunction: type 1 (mild deficiency), type 2 (functional abnormalities), and type 3 (severe deficiency) [12]. These inherited disorders typically show proportional reductions in both vWF antigen and activity. Treatment for AvWS involves managing the underlying condition, such as CKD or malignancies, while inherited vWF disorders are managed with vWF concentrates or desmopressin to correct vWF deficiencies and improve bleeding tendencies. Table 2 summarizes these differences in laboratory findings and clinical presentation.

**Table 2:** Comparison of laboratory findings and clinical presentation in acquired von Willebrand syndrome, inherited von Willebrand disease, and hemophilia. vWF: von Willebrand factor; AvWS: acquired von Willebrand syndrome.

Disorder	vWF antigen	vWF activity	Multimer pattern	Factor VIII level
Inherited vWF disorder	Reduced	Reduced	Type-specific (e.g., loss of high molecular weight in type 2A)	Low but proportional to vWF
AvWS	Variable	Disproportionately low	Abnormal, reflecting underlying conditions (e.g., loss of high molecular weight multimers)	Often disproportionately low
Hemophilia	Normal	Normal	Normal	Deficient (specific to type of hemophilia)

The following case of a patient with CKD who was diagnosed with AvWS underscores significant challenges: diagnosing AvWS among symptoms overlapping with CKD is complex and requires detailed testing to differentiate vWF abnormalities. Additionally, the short-lived response to desmopressin (DDAVP) complicates treatment due to CKD's impact on DDAVP efficacy, highlighting the need for careful management of both CKD and AvWS, along with ongoing monitoring to effectively address bleeding risks.

This occurrence of thrombocytopenia in this patient serves to illustrate the close association between CKD and coagulation disturbances. Thrombocytopenia is a common finding in patients with CKD, affecting up to one-fifth of the individuals [13]. The etiology of thrombocytopenia in CKD patients is not fully understood; however, the possible mechanisms include reduced production of thrombopoietin, accelerated platelet destruction, and impaired platelet formation [14]. Within the setting of AvWS, thrombocytopenia may further increase the risk of bleeding, which complicates the assessment and management of the patient.

The pathophysiology of AvWS in CKD is related to the abnormalities in vWF synthesis and activity. vWF is produced in endothelial cells and megakaryocytes of humans and is very vital in platelet adhesion and the formation of a firm platelet plug during coagulation [15].

In CKD, the uremic toxins and metabolic changes affect the structure and function of vWF, which in turn reduces the activity and impairs platelet adhesion [3]. Furthermore, patients with reduced kidney function will have a reduced clearance of vWF-cleaving proteases, which can further worsen the function of vWF [16].

The initial assessment should involve the review of the patient’s medical record, including their general health status, and standard laboratory investigations like the hematology and coagulation profiles. Nevertheless, as a result of this case, it has been seen that specialized testing is usually required to make a diagnosis of AvWS. Assays for vWF:Ag and vWF:Ac are considered the best tests for diagnosing AvWS, and low levels of the two are suggestive of the disease [17]. It should be noted that while renal dysfunction can affect the results of these tests, normal values may still be associated with the presence of AvWS in CKD patients [11].

The treatment of AvWS in the context of CKD differs from other situations due to the underlying renal dysfunction affecting hemostasis and treatment efficacy. In CKD-related AvWS, treatment often involves addressing the renal impairment itself, which can mitigate the AvWS symptoms. Desmopressin (DDAVP) is commonly used to increase vWF and factor VIII levels; however, its efficacy can be variable in CKD patients due to altered pharmacokinetics and the presence of uremic toxins that affect vWF function. In cases where DDAVP is ineffective or contraindicated, vWF concentrates or recombinant factor VIII may be used [11]. Additionally, dialysis can help reduce uremic toxins and improve hemostasis in CKD patients, indirectly benefiting AvWS management [18].

In contrast, AvWS in other contexts, such as malignancies or autoimmune diseases, may respond better to immunosuppressive therapy or treatment of the underlying condition. For example, rituximab or corticosteroids are often used in AvWS associated with lymphoproliferative disorders [19]. Indications for treating AvWS include significant bleeding, preparation for surgery or invasive procedures, and maintaining hemostasis in patients with a high risk of bleeding [20]. Monitoring involves regular assessment of vWF activity, factor VIII levels, and clinical evaluation for bleeding symptoms, with adjustments to treatment based on these findings [20].

The outcomes of the treatment in AvWS patients with CKD are not always predictable, so it is crucial to pay attention to the efficiency and tolerability of the chosen management plan. In this case, the patient’s bleeding symptoms were managed, while his platelet count returned to the normal range after treatment. However, the sustained management of AvWS in CKD patients may be associated with maintenance therapy and periodic assessment of the patient’s renal status and bleeding manifestations. Renal transplantation has also been shown to reduce AvWS in CKD patients, stressing the importance of kidney dysfunction in the development of this complication [11].

## Conclusions

This case report highlights an atypical manifestation of AvWS in a patient with CKD that led to unexpected thrombocytopenia and bleeding events. While CKD itself is not a direct cause of AvWS, it can contribute to its development by affecting vWF function and clearance. Therefore, AvWS should be considered in the differential diagnosis of bleeding disorders in CKD patients. Early identification, appropriate diagnostic procedures, and treatment of AvWS are crucial for managing bleeding risks in this population. Healthcare professionals should be vigilant for bleeding manifestations in CKD patients and consider AvWS as a potential diagnosis to ensure effective care.
